# Real-time Transcriptional Profiling of Cellular and Viral Gene Expression during Lytic Cytomegalovirus Infection

**DOI:** 10.1371/journal.ppat.1002908

**Published:** 2012-09-06

**Authors:** Lisa Marcinowski, Michael Lidschreiber, Lukas Windhager, Martina Rieder, Jens B. Bosse, Bernd Rädle, Thomas Bonfert, Ildiko Györy, Miranda de Graaf, Olivia Prazeres da Costa, Philip Rosenstiel, Caroline C. Friedel, Ralf Zimmer, Zsolt Ruzsics, Lars Dölken

**Affiliations:** 1 Max von Pettenkofer-Institute, Ludwig-Maximilians-University, Munich, Germany; 2 Gene Center and Department of Biochemistry, Ludwig-Maximilians-University, Munich, Germany; 3 Institute for Informatics, Ludwig-Maximilians-University, Munich, Germany; 4 Department of Molecular Biology, Princeton University, Princeton, New Jersey, United States of America; 5 School of Biomedical and Biological Sciences, Centre for Research in Translational Biomedicine, Plymouth University, Plymouth, United Kingdom; 6 Department of Medicine, University of Cambridge, Addenbrooke's Hospital, Cambridge, United Kingdom; 7 Institute for Microbiology, Immunology and Hygiene, Technical University, Munich, Germany; 8 Institute of Clinical Molecular Biology, Christian-Albrechts-University, Kiel, Germany; University of Southern California Keck School of Medicine, United States of America

## Abstract

During viral infections cellular gene expression is subject to rapid alterations induced by both viral and antiviral mechanisms. In this study, we applied metabolic labeling of newly transcribed RNA with 4-thiouridine (4sU-tagging) to dissect the real-time kinetics of cellular and viral transcriptional activity during lytic murine cytomegalovirus (MCMV) infection. Microarray profiling on newly transcribed RNA obtained at different times during the first six hours of MCMV infection revealed discrete functional clusters of cellular genes regulated with distinct kinetics at surprising temporal resolution. Immediately upon virus entry, a cluster of NF-κB- and interferon-regulated genes was induced. Rapid viral counter-regulation of this coincided with a very transient DNA-damage response, followed by a delayed ER-stress response. Rapid counter-regulation of all three clusters indicated the involvement of novel viral regulators targeting these pathways. In addition, down-regulation of two clusters involved in cell-differentiation (rapid repression) and cell-cycle (delayed repression) was observed. Promoter analysis revealed all five clusters to be associated with distinct transcription factors, of which NF-κB and c-Myc were validated to precisely match the respective transcriptional changes observed in newly transcribed RNA. 4sU-tagging also allowed us to study the real-time kinetics of viral gene expression in the absence of any interfering virion-associated-RNA. Both qRT-PCR and next-generation sequencing demonstrated a sharp peak of viral gene expression during the first two hours of infection including transcription of immediate-early, early and even well characterized late genes. Interestingly, this was subject to rapid gene silencing by 5–6 hours post infection. Despite the rapid increase in viral DNA load during viral DNA replication, transcriptional activity of some viral genes remained remarkably constant until late-stage infection, or was subject to further continuous decline. In summary, this study pioneers real-time transcriptional analysis during a lytic herpesvirus infection and highlights numerous novel regulatory aspects of virus-host-cell interaction.

## Author Summary

Cytomegaloviruses are large DNA viruses, which establish life-long latent infections, leaving the infected individual at risk of reactivation and disease. Here, we applied 4-thiouridine-(4sU)-tagging of newly transcribed RNA to monitor the real-time kinetics of transcriptional activity of both cellular and viral genes during lytic murine CMV (MCMV) infection. We observed a cascade of MCMV-induced signaling events including a rapid inflammatory/interferon-response, a transient DNA-damage-response and a delayed ER-stress-response. All of these were heavily counter-regulated by viral gene expression. Besides dramatically increasing temporal resolution, our approach provides the unique opportunity to study viral transcriptional activity in absence of any interfering virion-associated-RNA. Virion-associated-RNA consists of transcripts that are unspecifically incorporated into the virus particles thereby resembling the cellular RNA profile of late stage infection. A clear picture of which viral genes are expressed, particularly at very early times of infection, could thus not be obtained. By overcoming this problem, we provide intriguing insights into the regulation of viral gene expression, namely 1) a peak of viral gene expression during the first two hours of infection including the expression of well-characterized late genes and 2) remarkably constant or even continuously declining expression of some viral genes despite the onset of rapid viral DNA replication.

## Introduction

Herpesviruses are large DNA viruses which cause a broad range of disease ranging from the common cold sore to cancer. They all share the ability to establish a life-long, latent infection, leaving the infected individual at constant risk of reactivation and subsequent disease. The human cytomegalovirus (HCMV) poses a severe threat to immunocompromised patients and represents the most common infective cause of congenital disorders affecting about 1 in 1,000 newborns [1]. Like all herpesviruses, cytomegaloviruses (CMV) have co-evolved with their animal and human hosts for millions of years. During this time, they have mastered host-cell modulation to facilitate their needs and thus provide ideal tools to study many fundamental cellular processes.

Numerous signaling events are triggered during the first few hours of infection. As such, binding of CMV particles to the cell membrane and virus entry result in the activation of cellular signaling pathways, some of which, *e.g.* NF-κB signaling, play an important role in initiating lytic viral infection [2]–[4]. Concomitantly, viral pathogen-associated molecular patterns are recognized by pattern-recognition receptors, resulting in robust activation of an innate immune response. Virion-associated proteins as well as the advent of viral gene expression then counteract intrinsic and arising host cell defense [5]. Several high-throughput studies addressed the transcriptional response of the cell to lytic CMV infection by analyzing temporal changes in total RNA levels [3], [6]–[10]. These studies revealed lytic CMV infection altered the expression of numerous cellular genes involved in a variety of processes including inflammation, innate immunity, cell cycle progression, cellular metabolism and cell adhesion.

One of the earliest events upon entry of the viral DNA into the nucleus is the deposition of viral genomes at nuclear domain (ND10) bodies [11], [12]. This appears to be part of an intrinsic antiviral defense mechanism suppressing the expression of foreign DNA entering the nucleus [13]. In part, this is mediated by chromatin-remodeling enzymes recruited to these structures [14]–[16]. In HCMV infection, this intrinsic host defense is overcome by the viral tegument protein pp71 [17], [18] as well as the viral immediate early 1 (IE1) protein [19], [20]. In lytic murine cytomegalovirus (MCMV) infection, dispersion of ND10 bodies seems to be predominately mediated by the IE1 protein [21] (reviewed in [22]). In addition to disruption of ND10 body-mediated antiviral defense, the immediate-early proteins initiate the lytic replication cycle by facilitating the transcription of early genes [23], [24]. The latter then modulate host cell environment, disarm the arising immune response, and establish the viral replication machinery. Upon viral DNA replication, viral late gene expression is initiated, culminating in the production and release of infectious virus particles [25].

The analysis of *de novo* early viral gene expression has been substantially hindered by large amounts of so called ‘virion-associated RNA’, unspecifically bound by the virus particles and delivered to the newly infected cell [26]–[30]. Chromatin immunoprecipitation (ChIP) has thus been employed to study the kinetics of viral transcriptional activity by looking at markers of active and inactive chromatin associated with the viral promoters. Immediately upon infection of permissive fibroblasts (at ‘pre-IE’ times of infection, using low multiplicities of infection) HCMV genomes become associated with markers of repressed chromatin [31]. As infection progresses, the chromatin status of viral promoters reflects the cascade of viral immediate-early, early and late gene expression [32], [33].

Standard gene expression analysis (using total RNA) to study kinetics of transcriptional regulation has several limitations. Firstly, short-term changes in total RNA levels do not match changes in transcription rates but are inherently dependent on the RNA half-life of the respective transcripts [34]. This strongly favors the detection of up-regulation of short-lived transcripts, commonly encoding for transcription factors and genes with regulatory function. This, in turn, may result in substantial bias in downstream bioinformatics analyses. Secondly, the temporal resolution - particularly for down-regulated genes - is rather low due to the relatively long median RNA half-life (5–10 h) in mammalian cells [35], [36]. The same is true for detecting (viral) counter-regulation of cellular genes induced earlier in infection. Thirdly, alterations in RNA synthesis rates cannot be differentiated from changes in RNA decay rates. Finally, transcriptional activity of the incoming CMV genomes cannot be definitively studied due to the presence of virion-associated RNA introduced to the newly infected cells by the incoming virus particles [28], [37].

Recently, we developed an approach termed 4-thiouridine-(4sU)-tagging to purify newly transcribed RNA from total cellular RNA [34]. This is applicable to a broad range of organisms including vertebrates, drosophila and yeast [38], [39]. In short, cells are cultured in presence of 4sU resulting in metabolic thiol-labeling of newly transcribed RNA at a frequency of about one 4sU residue in 50 to 100 nucleotides [34]. After isolation of total cellular RNA, RNA-incorporated 4sU is thiol-specifically biotinylated. Labeled newly transcribed RNA is then efficiently purified from total RNA using streptavidin-coated magnetic beads. All three RNA fractions, *i.e.* total, newly transcribed and unlabeled pre-existing RNA, are suitable for quantitative RT-PCR (qRT-PCR), microarray analysis and next-generation sequencing [34], [40]–[43]. In the present study, we employed this approach to lytic murine cytomegalovirus (MCMV) infection of fibroblasts to study the real-time kinetics of cellular and viral gene expression using qRT-PCR, microarray analysis and RNA-sequencing (RNA-seq). We show that this approach circumvents all the caveats mentioned above, thereby providing intriguing new insights into cytomegalovirus host-cell modulation and regulation of viral gene expression.

## Results

### Establishment of 4sU-tagging for lytic MCMV infection

**Figure 1:**
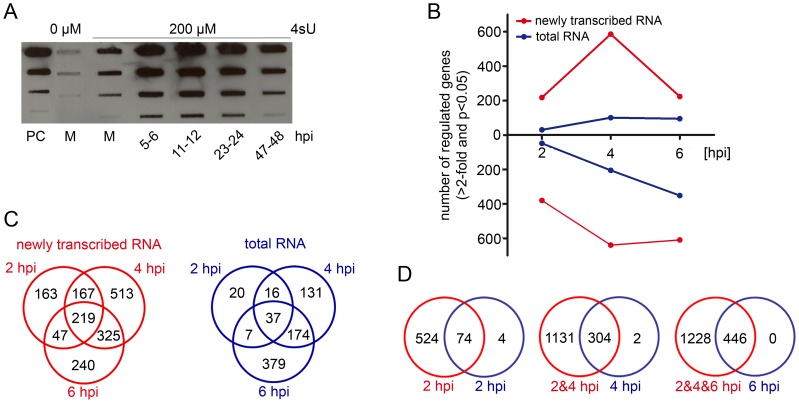
Establishment of 4sU-tagging for lytic MCMV infection. (**A**) Incorporation of 4sU throughout MCMV infection. Cells were infected with MCMV at an MOI of 10 and exposed to 200 µM 4sU for 1 h at different times of infection before total RNA was isolated. Thiol-specifically biotinylated RNA was subjected to dot blot analysis in 10-fold dilutions (1 µg down to 1 ng). A biotinylated oligonucleotide of 81 nt (PC, 100 ng down to 0.1 ng) was used to quantify 4sU-incorporation; M = mock control. (**B**)–(**D**) Comparison of genes identified to be regulated in newly transcribed RNA to genes regulated in total RNA. (**B**) Numbers of genes up- and down-regulated (>2-fold, p≤0.05) at different times of infection are shown for newly transcribed RNA and total RNA. (**C**) Venn diagrams of all genes regulated more then 2-fold in newly transcribed RNA and total RNA. (**D**) Venn diagrams showing genes regulated >2-fold in total RNA at 2, 4 and 6 hpi and in newly transcribed RNA at and prior to the indicated time point of infection; red = newly transcribed RNA, blue = total RNA.

Upon its addition to the cell culture medium, 4-thiouridine (4sU) is rapidly taken up by cells, phosphorylated and incorporated into newly transcribed RNA in a concentration-dependent manner [34]. To establish 4sU-tagging for MCMV infection, we first analyzed the effect of lytic MCMV infection on 4sU-incorporation. NIH-3T3 fibroblasts were infected with MCMV at a multiplicity of infection (MOI) of 10. At different times of infection, 200 µM 4sU was added to the cell culture medium for 1 h. Total RNA was prepared and subjected to thiol-specific biotinylation. 4sU-(biotin)-incorporation was quantified by dot blot (Figure 1A). At all times of infection, 4sU-incorporation was at least as efficient as in uninfected cells, ensuring efficient purification of newly transcribed RNA at all times of infection. Interestingly, from 5 to 24 hours post infection (hpi) the extent of 4sU-incorporation into cellular RNA was about 20-fold greater than in uninfected cells. By 47–48 hpi this had returned to levels found in uninfected cells. These data are consistent with increased transcriptional activity as well as enhanced nucleoside metabolism during lytic CMV infection [44].

Metabolic labeling of newly transcribed RNA with 4sU has negligible polar effects on eukaryotic cells [34], [45], [46]. To exclude gross adverse effects of 4sU-labeling on MCMV replication, we applied 1 h of 200 µM 4sU-treatment to NIH-3T3 cells at different times of infection. No effect of 4sU exposure on virus titers, determined at 48 hpi, was observed (Figure S1). We therefore decided to use 1 h of 200 µM 4sU in all following experiments.

### Kinetics of transcriptional activity of cellular genes during early MCMV infection

To detail transcriptional changes in host gene expression during early MCMV infection, we infected NIH-3T3 fibroblasts with MCMV at an MOI of 10 and labeled newly transcribed RNA from 1–2, 3–4 and 5–6 hpi. Three replicates of both total and newly transcribed RNA were subjected to Affymetrix Gene ST 1.0 arrays. After Robust Multichip Average (RMA) normalization, we identified all genes significantly regulated (p≤0.05) by at least 2-fold compared to uninfected cells in any condition. This resulted in the identification of 1,674 probe sets showing differential expression (Table S1a). With the exception of 4 genes, all differentially expressed genes were either exclusively up- or down-regulated during the first 6 h of infection. The number of genes with differential expression detectable in total RNA only represented 13% (at 2 hpi), 25% (at 4 hpi) and 54% (at 6 hpi) of those identified in newly transcribed RNA. As predicted, down-regulation only started to become detectable in total RNA with substantial delay, i.e. at 4 hpi (Figure 1B). Furthermore, a peak of MCMV-induced and rapidly counter-regulated gene expression was apparent in newly transcribed RNA at 3–4 hpi. This was invisible in total RNA. The overlap of differential gene expression detectable at different times of infection was substantially greater for newly transcribed RNA (Figure 1C). Notably, we found all genes induced or repressed by at least 2-fold in total RNA at 6 hpi to show concordant regulation in newly transcribed RNA (Figure 1D). In addition, only 3 probe sets showed more than 2-fold greater regulation in total RNA than in newly transcribed RNA (genes listed in Table S1b). Hence, the vast majority of differential gene expression during the first six hours of MCMV infection is the result of alterations in transcription rates and not due to changes in RNA decay rates. We therefore decided to focus all our subsequent analyses on newly transcribed RNA.

### 4sU-tagging details discrete functional gene clusters regulated with distinct kinetics

**Figure 2:**
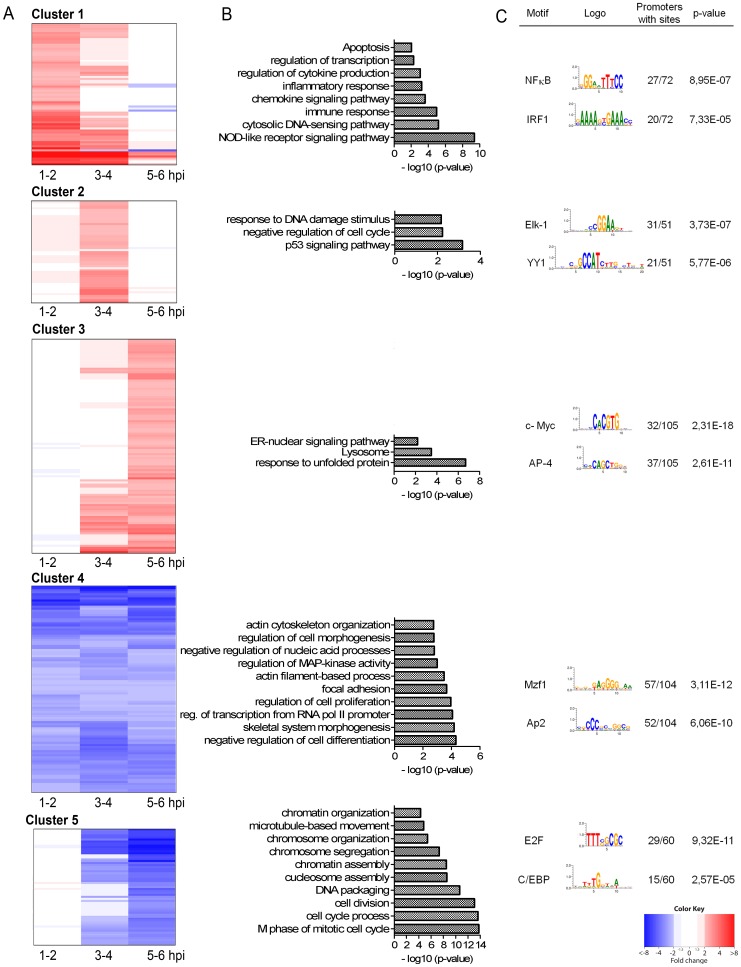
Gene expression kinetics define distinct functional clusters. (**A**) Heat-maps indicating the fold-changes are shown as matrices with rows representing genes and columns representing the time points post infection. Red represents up-regulation, blue down-regulation (>2-fold, p≤0.01) in newly transcribed RNA relative to uninfected cells. Ordering of genes in the heat-maps was determined using non-supervised hierarchical clustering. Shown are the 5 clusters of genes we identified. (**B**) All clusters are associated with distinct functional annotations. Enrichment analysis results of Gene Ontology ‘Biological Process’ terms and KEGG pathways are shown for each of the five clusters with the most significant (p≤0.01) categories displayed in the graphs as bars, sorted from bottom (most significant) to top. To reduce redundancy, similar terms are represented by the most significant and specific term. For complete list of functional annotations see Table S2a. (**C**) Specific transcription factor binding sites correlate with functional clusters. Shown are exemplary transcription factors with over-represented binding sites unique for the different clusters. For a complete list of over-represented transcription factor weight matrices see Table S2b. Illustrated are the transcription factor weight matrices, the percentage of promoters with sites and p-value.

Clustering genes based on >2-fold differences in regulation at different times of infection, we identified 5 clusters of genes characterized by distinct kinetic profiles (Figure 2A; for details and genes represented in each cluster see Table S1c and materials and methods). MCMV-induced genes peaked at 1–2 (Cluster 1), 3–4 (Cluster 2) or 5–6 hpi (Cluster 3). Of these, Cluster 2 was not detectable in total cellular RNA at all. Rapid and rather constant down-regulation was characteristic of genes in Cluster 4, while genes in Cluster 5 showed delayed down-regulation. These five clusters also became apparent when unsupervised clustering based on the changes in induction between 1–2, 3–4 and 5–6 hpi was performed (see Figure S2). To look for functional characteristics of these five gene clusters, we performed an enrichment analysis of Gene Ontology (GO) terms (biological process) and KEGG pathways. Interestingly, all five clusters were associated with distinct functional terms (see Figure 2B; for complete list of over-represented gene ontologies see Table S2a). Genes in Cluster 1 were involved in immune and inflammatory processes as well as apoptosis. Genes in Cluster 2 mainly played a role in p53 signaling and cell cycle progression. Delayed induction was observed for genes involved in the ER stress response (Cluster 3). Rapid and sustained down-regulation was observed for genes involved in cell proliferation and differentiation, focal adhesion as well as actin filament-based processes (Cluster 4). Finally, delayed down-regulation was characteristic of genes with a role in chromatin assembly and cell cycle processes (Cluster 5). Due to the delayed visibility of down-regulation in total RNA (see Figure 1B), Cluster 4 and 5 could only be differentiated using newly transcribed RNA. This approach thus allowed dissecting differential gene expression into discrete functional clusters regulated with distinct kinetics. These provided us with ideal templates to elucidate the underlying transcription factors and molecular mechanisms using *in silico* promoter analysis.

### Promoter analysis associates distinct transcription factors with each individual gene cluster

We performed promoter analysis on the five clusters to identify cellular transcription factors (TFs) involved in their regulation. Proximal promoter regions (PPR) ranging from −500 to +100 bp from the transcription start site (TSS) were analyzed for over-represented transcription factor binding motifs. While a number of transcription factor binding motifs were over-represented in the five clusters (for complete list and data see Table S2b), we observed distinct transcription factor binding sites to be uniquely over-represented in each of the individual clusters (see Figure 2C for exemplary TFs and Table S2b). These correlated very well with the functional annotations of the associated clusters. In Cluster 1, uniquely over-represented binding sites were found for NF-κB and IRF-1. NF- κB is one of the key players of the cellular immune response and it thus has important roles in the antiviral defense [47]. Infection with CMV results in an activation of this transcription factor [48], however, subsequently it is counter-regulated by the virus. In the case of MCMV this is mediated by the viral protein M45 [49], [50]. IRF-1 is an important factor in the antiviral IFN response and, like canonical type I interferon signaling, results in activation of promoters containing interferon stimulated response elements (ISREs). HCMV pp65 can inhibit activity of IRF-1 [51]. In addition, the MCMV M27 counteracts the interferon response by targeting Stat2 for degradation [52].

Elk-1 and YY1 are examples of transcription factors for which binding sites were over-represented in the cluster of genes (Cluster 2) showing a peak of induction at 3–4 hpi. Elk-1 is activated by the MAPK/ERK pathway, which is stimulated during HCMV infection [53]. YY1 is a DNA-binding transcription factor that acts as a repressor of some promoters and an activator of others [54]. It has been shown that YY1 can directly bind to the HCMV major IE promoter region and mediates repression of HCMV IE gene expression [55]. In latent MCMV infection, YY1 is also specifically recruited to the major immediate early protein (MIEP) promoter and might thus play a role in the control of latency and reactivation [56].

**Figure 3:**
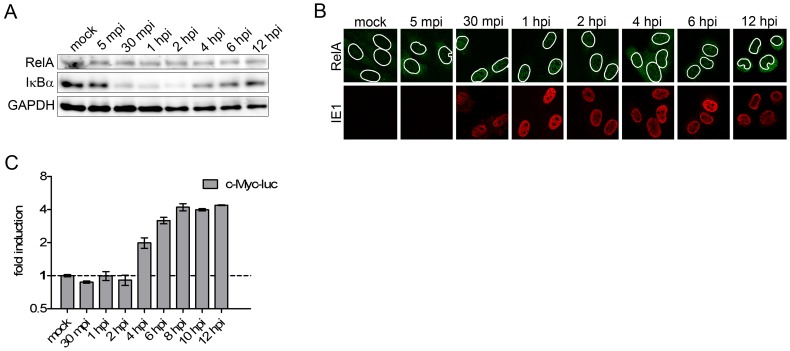
Validation of exemplary transcription factors. NIH-3T3 fibroblasts were infected with MCMV at an MOI of 10 for the indicated time points and lysates were prepared for western blot analysis (**A**), for immune staining (**B**) or luciferase assay (**C**). Western blot analysis was performed on samples prepared from uninfected and infected NIH-3T3 cells probed for RelA and IkBα (**A**). GAPDH was probed as loading control. For the immunofluorescence staining (**B**) cells were fixed and stained with the indicated antibodies; white circle indicating nucleus, nuclear dimensions were acquired by DAPI staining and its outline was overlaid into the shown channels; green, RelA; red, viral IE1. For the luciferase assays cells were transfected with a c-Myc-reporter construct (**C**) and infected 48 hours post transfection with MCMV at an MOI of 10. At the indicated times post infection, Firefly-Luciferase measurements were performed in triplicates. Shown is the mean +/− SD of a representative of three experiment; mpi = minutes post infection, hpi = hours post infection.

For Cluster 3, which contained genes induced with delayed kinetics (Figure 3C), we found over-represented binding sites for c-Myc and Ap-4. c-Myc is a proto-oncogene which drives cell cycle progression and apoptosis, whereas cellular differentiation and cell adhesion are negatively influenced [57]. Both, IE1 and IE2 proteins of HCMV were shown to be able to up regulate the c-Myc promoter and thus to increase c-Myc expression [58]. AP-4 was identified to be a direct transcriptional target of c-Myc [59].

In Cluster 4, genes had uniquely over-represented binding sites for Mzf1 and AP2. Mzf1 belongs to the Krüppel family of zinc finger proteins and plays a role in regulating transcriptional events during hemopoietic development and controls cell proliferation as well as tumorigenesis [60], [61]. Until now, nothing is known about a role of Mzf1 upon CMV infection. AP2 are a family of transcription factors which were shown to play role in apoptosis, cell-cycle control and proliferation as well as tumorigenesis [62]. Little is known about a role of AP2 in CMV infection.

Cluster 5 showed uniquely over-represented binding sites for E2F and C/EBP. E2F is a family of TFs which are involved in the regulation of S phase genes as well as DNA damage and apoptosis [63]. For E2F-1, Song and Stinski showed that HCMV increases its activity [64]. C/EBP belongs to the bZIP transcription factors and has important function in adipocyte differentiation, maintains energy homeostasis and regulates cell differentiation [65]. It can induce growth arrest by interacting with CDK2 and CDK4 and interacts with the heterodimer E2F-DP to inhibit cell growth [66], [67]. To date, little is known about its function in CMV infection, however, the MIEP promoter of CMV contains a binding site for this transcription factor [68].

Two exemplary TFs were chosen for further validation. As a proof-of-principle TF, we decided to look at NF-κB to see whether it's well-described rapid induction and counter-regulation during CMV infection [48], [49] would precisely reflect the transcriptional changes we observed in newly transcribed RNA under our experimental conditions. NF-κB-dimers of the NF-κB- (p105 and p100) and Rel-subfamily (c-Rel, RelB and RelA) are present in inactive IκB-bound complexes in the cytoplasm. IκK-mediated phosphorylation induces degradation of the inhibitor IkBα, enabling translocation of the NF-κB dimers to the nucleus and enhanced transcription of NF-κB target genes (reviewed in [69]). To look for degradation of the inhibitor IκBαduring the first 12 h of MCMV infection, we performed immunoblotting (Figure 3A). In addition, immunofluorescence analysis was performed to reveal the shift of RelA into the nucleus (Figure 3B). Results from both experiments demonstrated the kinetics of transcriptional regulation of genes in Cluster 1 to precisely mirror NF-κB activation, highlighting the ability of 4sU-tagging to detail real-time transcription factor activity.

In addition, we looked at a representative TF of Cluster 3, namely c-Myc. c-Myc forms a heterodimer with Max, followed by its binding to target genes [70]. Furthermore, phosphorylation of two amino acids at the NH2-terminal domain is important for transactivation of c-Myc [71]. Hagemeier et al. showed that HCMV IE1 and IE2 can transactivate the c-Myc promoter [58]. We performed luciferase assays using a c-Myc-specific reporter construct transfected into NIH-3T3 cells 48 h prior to infection to analyze c-Myc activation. Luciferase activity started to significantly increase at 4 hpi, matching the expression kinetics of genes in Cluster 3 (Figure 3C).

### Virus-mediated regulation and counter-regulation of host gene expression

We then addressed the role of viral gene expression in the regulation of each cluster using infection with UV-inactivated virus. To provide a more comprehensive picture, we extended the kinetics until 48 hpi. To this end, NIH-3T3 cells were infected with either wild-type (wt) or UV-inactivated virus. RNA was labeled for 1 h at different times of infection and newly transcribed RNA was purified. Transcription rates of exemplary genes of each functional cluster were determined in newly transcribed RNA using quantitative RT-PCR (qRT-PCR). This included NF-κB- (Cluster 1), interferon- (Cluster 1), DNA-damage- (Cluster 2) and ER-stress- (Cluster 3) induced genes as well as MCMV-repressed genes involved in the regulation of cell differentiation (Cluster 4) and cell cycle/chromatin organization (Cluster 5). The housekeeping gene Lbr (Lamin B receptor) was used for normalization.

**Figure 4:**
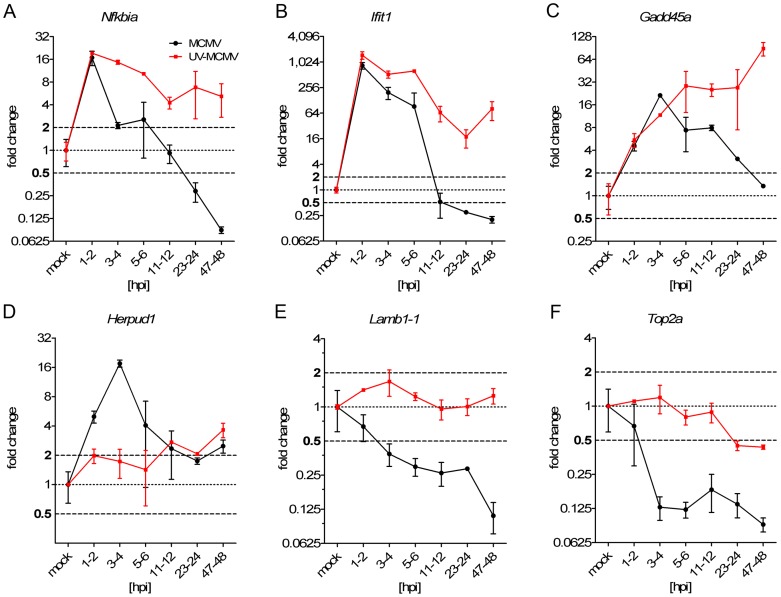
Identification of virus-specific regulation and counter-regulation using UV-inactivated virus. Quantitative RT-PCR was employed to measure transcription rates of exemplary genes of the five gene clusters (**A–F**). NIH-3T3 were infected with wild-type (wt) or UV-inactivated (1500 J, 15 min) MCMV (MOI of 10) for the indicated time points. Displayed are the fold-changes relative to uninfected cells normalized to Lbr. Fold-changes in between 0.5- and 2-fold were considered as non-regulated. Shown are the combined data (means +/− SD) of three independent experiments.

Cluster 1 contains both NF-κB- as well as interferon-induced genes. We thus chose NF-κBiα (NF-κB-inhibitor alpha), an NF-κB-induced negative regulator of the NF-κB response, as well as Ifit1 (Interferon-induced protein with tetratricopeptide repeats 1) for this analysis. Both NF-κBiα and Ifit1 were rapidly induced and counter-regulated by lytic MCMV infection (Figure 4A, B). Induction of both genes following infection with UV-inactivated virus was comparable to wt-MCMV infection, consistent with previous reports showing that viral gene expression is not required for induction of both NF-κB- and interferon-signaling. In both cases, however, counter-regulation was substantially delayed following infection with UV-inactivated virus. While counter-regulation of the NF-κB response is consistent with the MCMV M45 gene product efficiently targeting NF-κB- signaling [50], a viral gene product targeting the induction of the interferon response remains to be identified [72].

To monitor DNA-damage response-mediated signaling, we analyzed transcriptional activity of Gadd45a (Growth arrest and DNA damage-inducible protein A), a well characterized DNA damage- induced gene [73]. Consistent with our microarray data, qRT-PCR revealed the same slightly delayed induction at 3–4 hpi, followed by a more protracted counter-regulation than we observed for NF-κBiα and Ifit1. Interestingly, UV-inactivated virus also triggered the induction of Gadd45a with similar kinetics. This was, however, no longer counter-regulated, but continued to increase until 48 hpi (Figure 4C). For HCMV it has been described that IE1 increases p53 activity by phosphorylation through ATM, an important kinase in the DNA damage response [74]. While our data are indicative of counter-regulation of this response by an MCMV gene product, we cannot exclude that the induction and the enhanced response - at least in parts - reflects increased activation by the UV-damaged viral DNA.

For Cluster 3, expression of Herpud1 (Homocysteine-responsive endoplasmic reticulum-resident ubiquitin-like domain member 1 protein), a gene induced by endoplasmatic reticulum (ER) stress [75], was monitored. Delayed induction was observed, which was rapidly counter-regulated. Induction of Herpud1 was lost upon infection with UV-inactivated virus, consistent with viral gene expression being required for the induction of the ER stress response. In summary, these findings indicate that a so far unknown viral gene product counteracts the ER stress response provoked by viral gene expression (Figure 4D). For HCMV, this function is thought to be performed by the viral pUL38 protein [76].

For Clusters 4 and 5 we chose to monitor the transcription kinetics of Lamb1-1 (Laminin beta 1), an important extracellular matrix glycoprotein, and Top2α (Topoisomerase 2 alpha), which is involved in the control and alteration of the topologic states of DNA during transcription [77], [78]. Interestingly, consistent down-regulation of both genes was observed following wt-MCMV, but not UV-MCMV infection, indicating that viral gene expression is required for both their regulation (Figure 4 E, F).

In summary, these data highlight that all cellular signaling pathways we identified to be induced during early MCMV infection are rapidly counter-regulated by the virus later on. In contrast, down-regulation of defined cellular signaling pathways prevails and thus most likely represents an intentional action of the virus to facilitate its needs.

### Analysis of newly transcribed RNA allows studying viral gene expression in absence of virion-associated RNA

**Figure 5:**
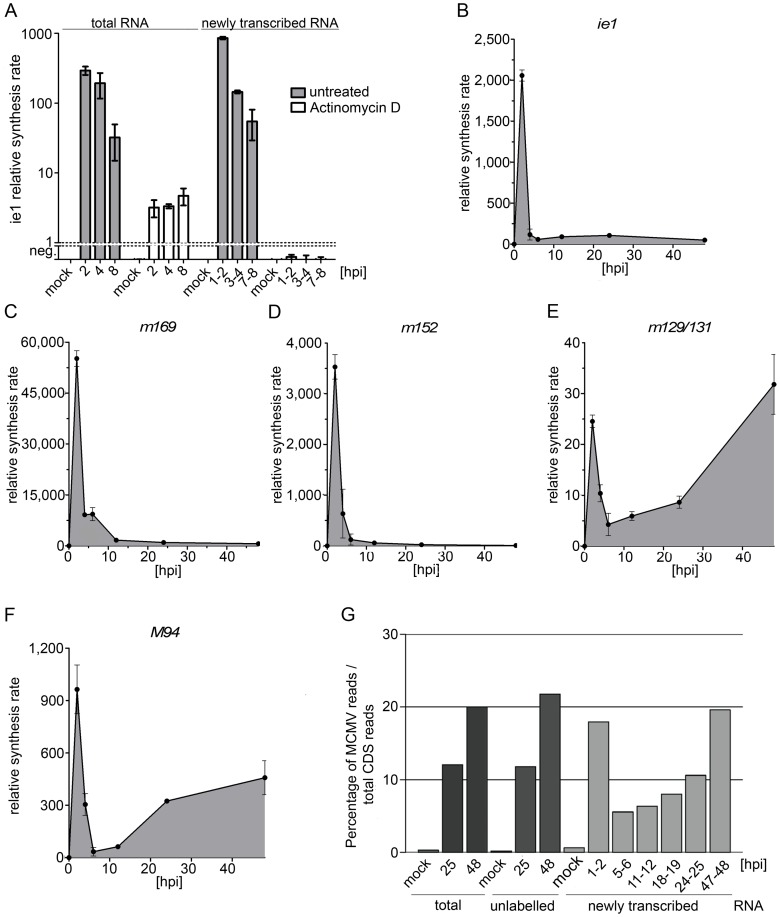
Real-time kinetics of viral gene expression. (**A**) 4sU-tagging allows efficient removal of viral DNA and virion-associated RNA. Newly transcribed RNA was labeled with 200 µM 4sU for 1 h in MCMV-infected NIH-3T3 cells at −1 to 0 (mock), 1–2, 3–4 and 7–8 hpi. As a negative control, Actinomycin-D was added to cells prior to infection to block transcription and thus 4sU-incorporation. Total RNA was isolated, treated with DNaseI and newly transcribed RNA was purified. qRT-PCR analysis was performed on newly transcribed RNA for viral ie1 and cellular Lbr. Shown are the combined data (means +/− SD) of three independent experiments. (**B–F**) Gene expression kinetics of exemplary viral genes. Shown are qRT-PCR measurements of newly transcribed RNA for ie1 (**B**), the early genes m169 (**C**) and m152 (**D**) as well as for the late genes m129/131 (**E**) and M94 (**F**). Synthesis rates were normalized to Lbr expression. Shown are the combined data (means +/− SD) of three independent experiments. (**G**) Contribution of viral transcripts to all coding sequence reads (CDS). RNA-seq was performed on newly transcribed, total and unlabeled pre-existing RNA samples (n = 1). Reads were mapped to both the cellular and viral transcriptome/genome. The contribution of viral reads to all CDS reads at different times of infection is shown.

Herpesvirus particles, like other herpesviruses, unspecifically incorporate and transfer large amounts of so called ‘virion-associated RNA’ to newly infected cells [26]–[30], [37]. This has substantially hindered detailed studies on the kinetics of viral gene expression during the first few hours of infection and in latency. 4sU-tagging allows the removal of virion-associated RNA and thus, the dissection of the regulation of viral gene expression during the initial phase of infection. To show that 4sU-tagged newly transcribed RNA fraction is indeed free of virion-associated RNA, we labeled newly transcribed RNA in MCMV infected NIH-3T3 cells from 1–2, 3–4 and 7–8 hpi in the presence and absence of the RNA polymerase II inhibitor Actinomycin D (Act-D). Act-D treatment inhibits RNA synthesis and thus prevents 4sU-incorporation into newly transcribed RNA. Following isolation of total RNA, we included a DNase digest prior to biotinylation to further remove viral DNA. Newly transcribed and total RNA samples were subjected to qRT-PCR analysis for the spliced viral ie1 gene and the cellular housekeeping gene Lbr. In total RNA, ie1 transcripts were detectable even in presence of Act-D, consistent with large amounts of virion-associated RNA delivered to the infected cells. However, in newly transcribed RNA virtually no ie1 and Lbr transcripts (below detection limit of our qRT-PCR assay) were detectable in presence of Act-D, consistent with the complete removal of virion-associated RNA (Figure 5A).

### 4sU-tagging reveals a peak of viral gene expression at 1–2 hpi, including immediate-early, early and even late gene expression

We then performed a comprehensive time-course analysis of transcriptional activity during lytic MCMV infection. To study relevant time frames, we first determined the kinetics of viral DNA replication in NIH-3T3 cells infected with wt-MCMV at an MOI of 10. Both qRT-PCR on M54, the catalytic subunit of MCMV DNA polymerase, and southern blot analysis of concatameric DNA revealed viral DNA replication to start at ∼15 hpi (Figure S3). We therefore decided to label newly transcribed RNA at 1–2 hpi, 3–4 hpi, 5–6 hpi, 11–12 hpi (prior to the onset of DNA replication), 24–25 hpi (first infectious virus particles starting to be released) and at 47–48 hpi (late stage infection). Following DNase digest and purification of newly transcribed RNA, this was subjected to qRT-PCR analysis for ie1 as well as two genes well characterized to be expressed with either early (m152 and m169) or late (m129/131 and M94) kinetics [79]–[81]. For the spliced late gene m129/m131 [82] we designed the qRT-PCR to span exon-exon junctions to further eliminate any residual risk of amplifying viral DNA or transcripts derived from the opposite DNA strand. To our great surprise, we found not only the two early, but also the two late genes to be well expressed during the first few hours of infection peaking at 1–2 hpi followed by a down-regulation at 5–6 hpi (Figure 5B–F). It is important to note that we could not detect any specific signals in any assay when qRT-PCR was carried out using non-reverse-transcribed samples or Act-D-treated samples (data not shown). This demonstrated the complete removal of viral DNA and virion-associated RNA from these samples. Expression of M94 was also observed when strand-specific cDNA synthesis was performed, matching the kinetics shown in Figure 5F (data not shown). In addition, agarose electrophoresis on m129/m131-PCR products confirmed a band of the predicted size, thereby excluding amplification of viral DNA or a transcript expressed from the opposite DNA strand (data not shown). Interestingly, qRT-PCR analysis on total RNA also revealed a transient, less prominent peak in viral transcript levels at 2 hpi for ie1 and m152 and at 4 hpi for m169 and m129/m131 (Figure S4). For ie1, Actinomycin-D treatment demonstrated that virion-associated RNA only comprised <5% of total RNA levels at 1–2 hpi (Figure 5A). Similar data were obtained for m152 (data not shown). Therefore, there appears to be at least a fraction of newly synthesized viral transcripts which are rather unstable (RNA half-life of ∼1 to 4 h).

A second unexpected finding was the dramatic drop of transcriptional activity of all viral genes starting 3–4 hpi. For ie1 and m169, transcription dropped >30-fold by 5–6 hpi (compared to 1–2 hpi) and then leveled off (Figure 5B, C). In contrast, transcription rates of m152 continued to drop exceeding 500-fold at 47–48 hpi (Figure 5D). Both late genes, *i.e.* m129/m131 and M94, showed a substantial increase in synthesis rates with the onset of viral DNA replication, consistent with their kinetic class. To rule out that our observations were simply caused by the high MOI, we repeated the experiment using a low MOI of 0.5 (Figure S5). As observed with high MOI, ie1 transcription had already peaked by 1–2 hpi. In contrast, transcription rates of both the two early and late genes had not peaked and were now peaking at 3–4 hpi (see Figure S5). Nevertheless, transcription rates of all five genes significantly dropped by 5–6 hpi. These data indicate that transient expression of viral late genes during the first few hours of infection is not an artifact of high MOI.

### RNA-seq reveals rapid silencing of viral gene expression after the initiation of early gene expression

**Figure 6:**
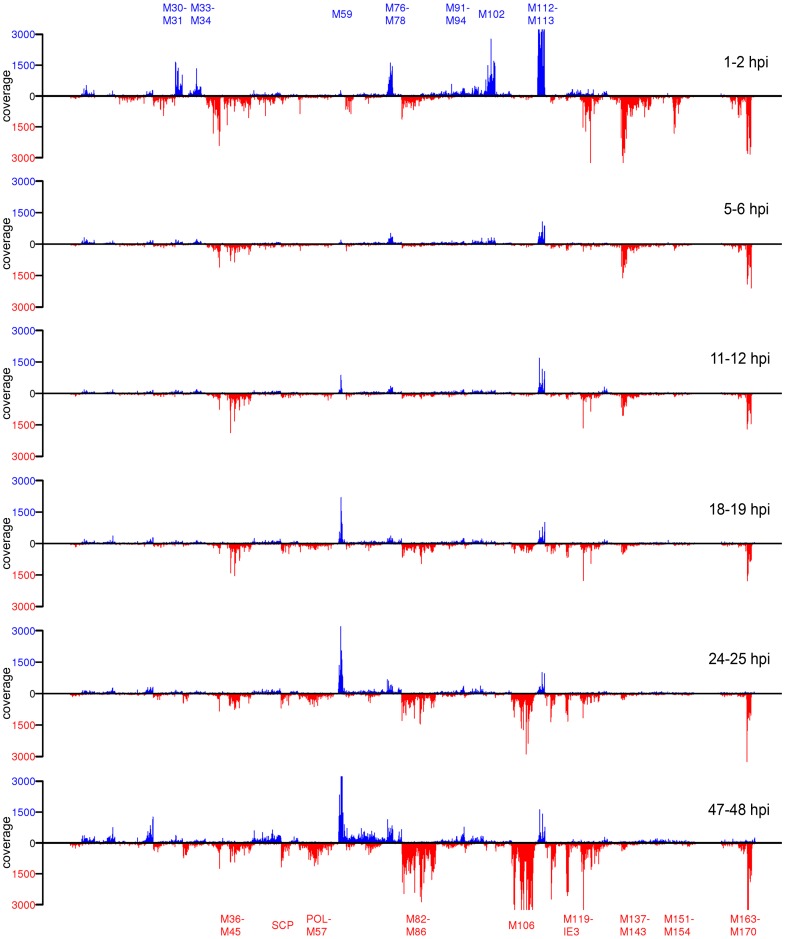
Analysis of MCMV gene expression by RNA-seq. Shown are the coverages of all viral genes across the MCMV genome obtained by RNA-seq on newly transcribed RNA from uninfected cells, 1–2 hpi, 5–6 hpi, 11–12 hpi, 18–19 hpi, 24–25 hpi and 47–48 hpi, normalized to the total number of reads mapped to mouse exons and annotated MCMV coding sequences. Genomic positions of exemplary genes as well as concordantly regulated gene clusters are marked; SCP = small capsid protein (m48.2), POL = polymerase (M54). Blue represents transcripts matching the direct DNA strand and red the complementary DNA strand.

To confirm these observations at the whole transcriptome level, we repeated the experiment described above and subjected newly transcribed RNA samples from 7 time points (mock, 1–2, 5–6, 11–12, 18–19, 24–25 and 47–48 hpi) to next-generation sequencing. In addition, we included samples of total and pre-existing RNA (mock, 25 hpi and 48 hpi). We obtained between 16 and 42 million reads per sample, which were mapped to the mouse transcriptome, mouse genome, MCMV predicted coding sequences and the MCMV genome in the respective order (for details on read numbers and mapping statistics see Table S4a). As expected, introns were substantially over-represented in newly transcribed RNA (Figure S6 and Table S4a) reflecting the substantially greater contribution of immature, unspliced nascent transcripts [41], [43]. When considering only coding sequences, viral transcripts accounted for ∼20% of all reads in total RNA at 48 hpi and in newly transcribed RNA at 47–48 hpi (Figure 5G). Interestingly, the extent of viral gene expression in newly transcribed RNA at 1–2 hpi also accounted for ∼15% of all reads, dropping to around 5% of all reads at 5–6 hpi. This corroborates our qRT-PCR finding of a burst of viral gene expression at 1–2 hpi, but also highlights that not all viral genes expressed at 1–2 hpi are subject to the same massive down-regulation we observed for m152. A closer look at the distribution of transcription rates across the whole viral genome (direct and complementary strand) revealed viral gene expression arising from multiple loci at 1–2 hpi (Figure 6). By 5–6 hpi, transcription rates of many, but not all, viral genes dropped substantially (for details see Table S4b). With the onset of viral DNA replication, late gene expression was initiated, accounting for the increasing number of viral reads at 24–25 and 47–48 hpi (although of shifted viral gene subsets compared to 1–2 hpi). At late stages of infection, transcription rates of viral genes stabilized, reflected by the continuous accumulation of the respective viral transcripts in both total and unlabeled pre-existing RNA (Figure S7).

## Discussion

In this work, we first employed 4sU-tagging combined with microarray analysis to study the dynamic changes in transcriptional activity of NIH-3T3 fibroblasts during early MCMV infection. Interestingly, we found virtually all changes in total cellular RNA to be matched by concordant changes in newly transcribed RNA, indicating that alterations in RNA decay rates do not substantially contribute to differential gene expression during early phase of MCMV infection of fibroblasts. This is consistent with previous reports showing that alterations in RNA decay rates do not seem to provide a major contribution during the first 3 h of the response of fibroblasts to type I and II interferons [34] or of dendritic cells to lipopolysaccharides [41]. It is important to note that the high MOI we employed facilitated the initiation of a fast, contemporaneous infection, crucial to dissect the temporal cascade of rapid transcriptional changes during lytic MCMV infection. This approach revealed extensive regulation, which remained undetectable in total cellular RNA. These elements of the host response to lytic infection are of particular interest because they are likely to be subjected to rapid viral counter-regulation. Analysis of newly transcribed RNA combined with the use of UV-inactivated virus detailed such rapid viral counter-regulation for inflammatory-, interferon-, DNA-damage- and ER-stress-induced changes. While numerous MCMV proteins have been shown to counteract the consequences of the induced ER-stress response, *i.e.* the induction of stress-induced natural killer cell activating ligands (reviewed in [83]), little is known about the counter-regulation of ER-stress signaling itself. The same is true for the very transient DNA-damage response we observed. The rapid counter-regulation in transcriptional activity revealed by newly transcribed RNA implies the existence of novel viral factors targeting these important cellular processes. Furthermore, our approach will now allow screening of large deletion mutants for the responsible viral genes.

Analysis of newly transcribed RNA revealed delayed down-regulation of genes involved in chromatin modification as well as down-regulation of genes involved in cell proliferation and actin filament-based processes. Within a few hours of infection, MCMV-infected cells show a profound cytopathic effect. The underlying molecular events remain to be elucidated. It is tempting to speculate that transcriptional down-regulation of genes involved in actin filament-based processes and cell adhesion, which we found to be consistently down-regulated as early as 1–2 hpi, contributes to this phenomenon.

As we exemplified for both NF-κB and c-Myc, changes in transcriptional activity (as detectable in newly transcribed RNA) directly mirror the changes in the activation status of the involved transcription factors. In addition, the ability to group the large number of differentially regulated genes (as usually identified when analyzing total RNA changes) into well-defined functional clusters of genes (regulated with distinct kinetics) further aids the subsequent success of *in silico* promoter analyses. Our approach thus provides an ideal mean to obtain insights into the molecular mechanisms/transcription factors involved. It is important to note that most of the transcription factors, implicated by our promoter analysis, have already been associated with the functional annotations of the respective gene clusters. In addition, many of the transcription factors specifically over-represented in the clusters (see Table S2b) were consistent with published data on their functional role in lytic CMV infection.

Of interest, E2F-sites were significantly over-represented in genes repressed with delayed kinetics (Cluster 5). The E2F family consists of both activating and repressing transcription factors (for review see [84], [85]). The activating E2F family members, E2F-1, -2 and -3, are important for the transactivation of target genes involved in G1/S transition and apoptosis. E2F-4 and -5 predominantly have repressive functions, mediating cell-cycle exit and cell differentiation. E2F-6, -7 and -8 also act as transcriptional repressors, but are less well characterized. A caveat of this is that the DNA-binding sites of the E2F-family members cannot be differentiated from each other by bioinformatics means, highlighting the complexity of this regulation. Therefore, the repression of genes in Cluster 5 could either be mediated by repression of activating E2F-family members (E2F-1,-2 and -3) or activation of repressive family members like E2F-4 and -5. Interestingly, the most significantly associated gene ontology for Cluster 5 was not ‘G1/S-phase of the mitotic cell cycle’, as would have been expected in case of E2F-1-associated regulation, but ‘M-phase of the mitotic cell cycle’. Recently, the LIN complex (LINC), which involves the repressive E2F-4 family member, was shown to selectively repress genes involved in G2/M phase [86]. A closer look at the genes of Cluster 5 revealed the presence of numerous genes reported by this and other work [87] to be key marker-genes repressed by the LINC complex (which involves E2F-4). These included Survivin (Birc5), Cyclin B1, Aurkb, Espl1 and Bub1. It is thus tempting to speculate that the repression of genes in Cluster 5 is mediated not by repression of activating E2F-family members, but by activation of repressive E2F-family members (involving LINC). Ongoing work seeks to clarify the role of E2F-family members in this regulation. Lytic HCMV infection has been shown to result in E2F-1 activation by rapid degradation of the under-phosphorylated form of pRB by the HCMV protein pp71 [88], [89] and to increase E2F-1 responsive genes [64]. Of note, we did not observe over-representation of E2F sites in any of the MCMV-induced gene clusters. While HCMV can only induce lytic infection in G0/G1 phase, MCMV can also efficiently replicate in cells that have passed through S phase by arresting them in G2 [90]. Differences in shifting cell populations may thus account for the lack of E2F-1 over-representation in our MCMV-induced gene clusters.

4sU-tagging provides the unique opportunity to study the regulation of viral gene expression in real-time without the interference of virion-associated RNAs. Employing 4sU-tagging combined with qRT-PCR and RNA-seq to lytic MCMV infection, we report on three surprising findings. 1) A peak of viral gene expression including expression of immediate-early, early and even well-characterized late genes at 1–2 hpi at both high and low MOI. 2) The rapid suppression of all three classes of viral gene expression by 5–6 hpi. 3) Very constant levels of viral gene transcription rates (e.g. for ie1 and m169) or even continuously increasing suppression (e.g. m152), despite the onset of extensive viral DNA replication.

Both qRT-PCR and RNA-seq on newly transcribed RNA revealed a peak of transcriptional activity of viral genes at 1–2 h of lytic MCMV infection, to an extent only reached again at late stages of infection (47–48 hpi). We were surprised to observe that this even included transcription of well-characterized late genes, e.g. m129–131 and M94. Rigorous controls excluded DNA contamination, virion-associated RNA and gene expression from the opposing DNA strand. Furthermore, this transcriptional activity was also observed at low MOI. In this case, however, the peak of both early (m152 and m169) and late (M94 and m129/131) gene expression slightly shifted from 1–2 hpi to 3–4 hpi, thus separating this from the peak of immediate-early gene expression, which still had already peaked at 1–2 hpi. Although even our low MOI infection will still have resulted in multiple virus particles entering the cells, this shift strongly argues against ‘leaky’ promoters being responsible for this late gene expression. It rather highlights a role of virus-mediated regulation (most likely mediated by IE- or viral tegument-proteins) in facilitating this part of viral gene expression. It is important to note that this early burst in ‘late’ transcripts at 1–2 hpi does not generate defective or partially processed transcripts. Transcripts were both poly-adenylated (cDNA synthesis for qRT-PCR was performed with oligo-dT primers) and fully spliced (m129-m131). Nevertheless, it is still unclear whether the respective transcripts are actually translated. Recent reports indicate regulation occurring at the level of translation to provide a major contribution to overall regulation of mammalian gene expression [42]. It is tempting to speculate that the virus uses additional, so far undisclosed mechanisms, to regulate its gene expression at post-transcriptional level. Of note, previous studies already postulated a role of post-transcriptional regulation in HCMV [91] and expression of some HCMV genes has already been shown to be regulated at post-transcriptional level [92], [93].

Interestingly, the peak of viral gene expression we observed at 1–2 hpi was followed by a rapid drop in transcription rates by 6–12 hpi. We were surprised to see this rapid down-regulation of early gene expression. Interestingly, this was sustained or even exaggerated at late times of infection. What is the cause of this down-regulation? Chromatin modifications of the viral genome are well known to play an important role during productive CMV infection (reviewed in [94]). The ND10 body-associated protein Daxx is known to rapidly repress transcription of incoming viral genomes by inducing repressive chromatin modifications around the HCMV major immediate-early promoter (MIEP) within 3 hpi. Dependent on the MOI of infection, the virus is able to overcome this repression. This results in the inhibition/reversion of HDAC-mediated repression of the viral genomes present in the nucleus and initiates the expression of early genes [21], [95]. It is important to note that the peak of early gene expression should thus only occur after ND10 body-mediated repression has already been efficiently disrupted. Our data is fully consistent with this hypothesis. While we observed both immediate-early and early gene expression to peak or already have peaked at 1–2 hpi following high MOI (consistent with rapid disruption of ND-10 bodies), low MOI resulted in a visible delay between the peak of immediate-early and early gene expression. This is consistent with a delayed dispersion of ND-10 body-mediated repression of early gene expression at low MOI. Of note, we observed repression of viral gene expression after the peak of early gene expression, i.e. after ND-10 bodies have already been dispersed. Therefore, this suppression, which we observed by both qRT-PCR and RNA-seq to occur in between 6 and 12 hpi, is unlikely to be due to the intrinsic antiviral defense known to be mediated by ND-10 bodies.

Interestingly, transcription of some genes, *e.g.* ie1 and m169 substantially dropped and then continued at low level despite the onset of viral DNA replication (i.e. a rapid >100-fold increase in viral DNA load). The most likely explanation for this observation is that the DNA architecture of *de novo* synthesized viral genomes does not support transcription of (at least) some viral genes. On the other hand, it is tempting to speculate that these differences in chromatin structure play an important role in initiating viral late gene expression. Interestingly, transcription rates of some viral genes, *e.g.* m152, continued to drop (exceeding 500-fold compared to 1–2 hpi) until 47–48 hpi. These observations highlight the importance of transcription factors in the regulation of viral gene expression. It is tempting to speculate that the early burst of transcriptional activity is due to activation of specific cellular transcription factors, which are transiently activated following virus entry but can only initiate viral gene expression (but for the IE genes) once ND-10 body-mediated repression has been overcome. Most likely, their subsequent de-activation by 5–6 hpi is responsible for the subsequent drop in transcription of viral genes we observed. It may result from cellular or viral mechanisms. With the onset of viral DNA replication these or other transcription factors are again activated culminating in viral late gene expression. Activation and repression of these transcription factors will also influence the expression of the cellular genes they govern. The kinetics of viral gene expression during the first few hours of infection best matches those of cellular genes within the Clusters 1 and 2. This is consistent with the well described presence of binding sites for NF-κB and even interferon stimulated response elements (ISRE) in many viral promoters [96], [97]. The changes in transcription factor activity following the advent of viral DNA replication are less well understood. 4sU-tagging now allows us to properly study the changes in cellular gene expression following the onset of viral DNA replication. Correlating these with the changes in viral gene expression will substantially enhance our understanding of how these viruses modulate the host-cell machinery for their own needs and pinpoint novel targets for antiviral intervention.

## Materials and Methods

### Cell culture and virus infection

Murine NIH-3T3 fibroblasts were cultured in DMEM (Gibco) supplemented with 5% fetal calf serum. Cells were seeded overnight to 80% confluence followed by infection with BAC-derived MCMV Smith strain. Infection was performed at an MOI of 10 using centrifugal enhancement (30 min, 2000 rpm) or an MOI of 0.5. The time point after centrifugation was marked as time point ‘0 min’ in all experiments. To block RNA polymerase II transcription, Actinomycin-D (Sigma) was used at a final concentration of 5 µg/ml. UV irradiation of virus stocks was performed with 1500 J/m^2^ UV light using a UV-Crosslinker (Vilber Loumart). Standard plaque assays were performed as described [98] to analyze the influence of 4sU on productive virus infection and to confirm the efficiency of UV-inactivation.

### Metabolic labeling and purification of newly transcribed RNA

RNA labeling was started by adding 200 µM 4-thiouridine (4sU, Sigma) to cell culture media for 1 h at different times of infection. At the end of labeling, total cellular RNA was isolated using Trizol reagent (Invitrogen). Biotinylation and purification of 4sU-tagged RNA (newly transcribed RNA) as well as dot blot analysis were performed as described previously [34]. For all samples subjected to qRT-PCR analysis, DNase I (Fermentas) treatment was performed on total RNA according to the manufacturer's instructions before biotinylation. RNA was recovered using the RNeasy Mini Kit (Qiagen).

### Reverse transcription and quantitative PCR

Reverse transcription was carried out in 20 µl reactions using Superscript III (Invitrogen) and Oligo-dT primers (Invitrogen) following the manufacturer's instruction. Samples were diluted 1∶5 with H_2_O before performing qRT-PCRs on a Light Cycler (Roche Molecular Biochemicals) as described in Dölken et al. [34]. Relative quantification was performed in relation to uninfected controls normalized to the housekeeping gene Lbr (Lamin B receptor). Primers were designed using the online Roche Universal Probe Library primer design tool spanning exon-exon junctions. All primers are listed in Table S3.

### Microarray analysis, data processing and statistical analysis

For the microarray analysis, 200 ng RNA of each sample was amplified and labeled using the Affymetrix Whole-Transcript (WT) Sense Target Labeling Protocol without rRNA reduction. Affymetrix GeneChip Mouse Gene 1.0 ST arrays were hybridized, washed, stained, and scanned according to the protocol described in WT Sense Target Labeling Assay Manual. Microarray data were assessed for quality and normalized with RMA. All microarray data are available at Gene Expression Omnibus (GEO) record GSE35919.

### Quality control, normalization, filtering, statistical testing

Data were analyzed using R and Bioconductor [99]. Only „present“ genes (*i.e.* expression values greater than 20 in at least 2 out of the total number of arrays for each RNA type) were included in downstream analysis. In the total RNA data set, n = 9,022 genes passed this filter; in the newly transcribed RNA data set, n = 9,399 genes passed. To better compare fold-changes between the total and newly transcribed data, the set union of genes (n = 10,071) was used. Differentially expressed genes were identified separately for the total RNA and newly transcribed RNA data sets using the LIMMA package. Differential expression was defined as having an estimated fold-change of at least 2 (calculated as the fold-change of the average expression in the triplicate measurements after infection compared to uninfected status) and a p-value smaller than 0.05 (adjusted for multiple testing using the Benjamini and Hochberg method [100]).

### Grouping of differentially expressed genes into co-regulatory modules and functional annotations

Newly transcribed transcripts, which showed differential expression in at least one condition, were grouped into clusters based on their fold-changes in newly transcribed RNA upon MCMV infection. Five clusters were defined (for gene lists see Table S1c). Briefly, cluster 1 contains rapidly induced genes (>2-fold at 1–2 hpi) which are >2-fold counter-regulated by 3–4 hpi. Cluster 2 comprises genes induced with slightly delayed kinetics (<2-fold induction at 1–2 hpi, but >2-fold at 3–4 hpi) showing >2-fold counter-regulation by 5–6 hpi. Cluster 3 comprised genes which were not induced at 1–2 hpi (<1.41-fold induced = 2^0.5^) but >2-fold induced by 5–6 hpi and not counter-regulated >2-fold by 5–6 hpi. Consistently down-regulated genes (>2-fold at 1–2, 3–4 and 5–6 hpi, with <1.41-fold variation in the extent of repression in between 1–2 and 5–6 hpi) define cluster 4. Finally, Cluster 5 comprises all genes not repressed >1.41-fold at 1–2 hpi but repressed >2.82-fold (2^1.5^) at 5–6 hpi. It is important to note that the criteria and cut-offs for these cluster were chosen empirically trying to keep the criteria simple. This resulted in cluster containing 50–100 genes thus providing solid templates for the down-stream bioinformatic analyses. Enrichment analysis of Gene Ontology ‘Biological Process’ terms and KEGG pathways was performed for each cluster using the DAVID bioinformatics analysis suite (http://david.abcc.ncifcrf.gov/; release 6.7) [101].

### Identification of over-represented transcription factor binding sites (TFBSs) in promoter regions

For all probe sets with a mapped EnsEMBL ID, the core promoter sequence (−500 to +100 bp relative to the transcriptional start site (TSS)) was retrieved using the Regulatory Sequence Analysis Tools (RSAT; http://rsat.ulb.ac.be/) *retrieve EnsEMBL seq* function. In case of alternative transcripts the most 5′ TSS was chosen. Over-represented TFBSs for each cluster were predicted using Transcription Factor Matrix (TFM) Explorer (http://bioinfo.lifl.fr/TFM/TFME/; release 2.0) [102]. Weight matrices modeling putative TFBSs were taken from TRANSFAC (version 6.0 public; vertebrate matrices only). P-value thresholds to define locally over-represented TFBSs were set to 0.0001 and 0.00001 for clusters of less or more than 100 genes, respectively.

### Immunoblotting and immunofluorescence

For immunoblotting, NIH-3T3 infected with MCMV were harvested in 500 µl cell lysis buffer (62.5 mM Tris, 2% SDS, 10% glycerol, 6 M urea, 5% β-mercaptoethanol, 0.01% bromophenol blue, 0.01% phenol red) at several time points of infection. Following heat denaturation (95°C, 5 min), 50 µl of the lysates were subjected to SDS-PAGE. Proteins were transferred to a nitrocellulose membrane (Schleicher & Schuell) using a semidry blotter (Peq-Lab) (2 h, 400 mA). Membranes were blocked in PBS +2.5% milk powder and subsequently incubated with primary antibody (o/n, 4°C). After incubation with horseradish peroxidase-conjugated secondary antibody (1 h, RT) the proteins were visualized by the ECL system (Perkin Elmer) in the Fusion FX device (Vilber Lourmat).

For indirect immunofluorescence NIH-3T3 cells were seeded onto fibronectin-coated glass cover-slips in 24-well plates and infected with MCMV. At various time points of infection, cells were fixed with 4% paraformaldehyde in DPBS (w/v) for 10 min at 37°C. The fixative solution was replenished twice with DPBS and the cells were permeabilized for 10 minutes with a solution of 0.1% Triton X-100 in DPBS. After extensive washing with DPBS, the cells were blocked using 3% (w/v) BSA in DPBS (blocking solution) for 1 h at room temperature (RT). Primary antibodies were applied in blocking solution and incubated with the cells at RT for 1 h followed by three DPBS washing steps and 1 h incubation at RT with 1∶1,000 dilutions of Alexa Fluor-conjugated, specific secondary antibodies (Invitrogen) in blocking solution. After a final washing step with DPBS, the preparations were mounted on glass slides with Prolong Gold including DAPI (Invitrogen) and analyzed using an LSM 710 (Zeiss) confocal laser scanning microscope with 405 nm, 488 nm and 561 nm laser excitation and appropriate filter sets.

The following primary antibodies were used for immunoblotting and -fluorescence: mouse anti-IE1 (CROMA101; kindly provided by S. Jonijic, University of Rijeka, Rijeka, Croatia), rabbit anti-GAPDH (mAbcam 9484) from Abcam, rabbit anti-RelA (A) and mouse anti-IκB-α (H4) from Santa Cruz Biotechnology.

### Luciferase assays

To monitor the activity of c-Myc-regulated signal transduction pathway, cells were transfected in a 6-well format using the Cignal c-Myc-Reporter (luc) Kit from Qiagen. 6 hours post transfection cells were seeded in 96-well plates (10,000 cells/well). 48 hours after seeding, the transfected cells were infected with MCMV (n = 3). At various times of infection, cells were lysed in 100 µl lysis buffer and luciferase firefly activity was determined according to the manufacturer's (Promega) instructions.

### Next Generation Sequencing

RNA was subjected to WTAK library construction to generate transcriptomic fragment libraries (50 bp, SOLiD (Life Technologies, Foster City, CA, USA) Total RNA-seq Kit V3) that preserve strandedness information of the reads. Molecular barcoding was used in order to pool several libraries in a single sequencing reaction according to the manunfacturer's protocol. Sequencing was performed using the SOLiD 3 system (Life Technologies). Potential sequencing errors were corrected using the SOLiD Accuracy Enhancement Tool (solidsoftwaretools.com/gt/project/saet). All sequencing data are available at Gene Expression Omnibus (GEO) at GSE35833.

### Short-read alignment, MCMV read coverage

Between 26 and 42 million 50 nt reads were obtained per sample (Table S4a). Reads were aligned in a 4-step process using the Bowtie alignment program. First, all reads were aligned to mouse transcripts (Ensembl version 63). Remaining unaligned reads were aligned to the mouse genome (mm9, NCBI Build 37). Remaining reads were aligned to MCMV coding sequences and finally to the full MCMV genomic sequence. Reads with ambiguous base calls, non-unique alignment positions or more than 4 mismatches were discarded. Reads were classified as exon-exon and exon-intron junction reads, respectively, if they overlapped an exon-exon or an exon-intron junction by ≥1 nt (Table S4a). MCMV genome coverage was scaled before plotting (for Figure 6 and S7). Scaling factors were derived using DESeq based on reads aligned to mouse exons. RPKM values of individual MCMV genes are included in Table S4b).

## Supporting Information

Figure S1 **No effect of 1 h 200 µM 4sU treatment on virus replication.** NIH-3T3 fibroblasts were infected with MCMV at an MOI of 10 for 48 h. Samples were exposed to 200 µM 4sU for 1 h at different time points of infection or left untreated. Supernatants were harvested and titrated at 48 hpi. Shown are the means +/− SD of three biological replicates.
(TIF)

Figure S2 **Cluster of all differentially regulated genes in newly transcribed RNA.** Heat-map indicating fold-changes with rows representing genes and columns representing time points post infection. Red represents up-regulation, blue down-regulation (>2-fold, p≤0.05) in newly transcribed RNA relative to uninfected cells. Ordering of genes was determined using non-supervised hierarchical clustering across two conditions: regulation at 3–4 hpi *minus* 1–2 hpi and regulation at 5–6 hpi *minus* 1–2 hpi (using log_2_-values of fold-changes compared to uninfected cells). Clustering was performed separately for induced and repressed genes. Gray scales depicted on the left mark genes belonging to the corresponding clusters described in Figure 2A.
(TIF)

Figure S3 **Temporal kinetics of MCMV replication.** NIH-3T3 cells were infected with MCMV at an MOI of 10 and DNA was isolated at various times of infection using the DNeasy Blood & Tissue Kit (Qiagen) according to the manufacturer's instructions. Prior to amplification, extracted DNA was digested with *Pae*I for 1 h, 37°C followed by heat inactivation. (**A**) TaqMan qRT-PCR was performed in triplicates for MCMV M54 and cellular Lbr using the ABI Prism 7700 sequence detector (Applied Biosystems) as described [103]. Synthesis rates were normalized to Lbr. Shown are the means +/− SD of three independent experiments. (**B**) Southern Blot analysis was performed as described in Popa et al. [104]. Shown is the detection of different genome fragments in DNA isolated from NIH-3T3 cells at various times of infection. MCMV BAC-DNA (BAC) served as a negative control. The 3 kb fragment serves as a measurement of genomic load, the 2 kb fragment for concatameric/circular DNA and the 1 kb fragment for cleaved genomic DNA. Due to its circular form, digested BAC DNA produces only two of the three fragments.
(TIF)

Figure S4 **Gene expression kinetics of exemplary viral genes in total RNA.** Shown are qRT-PCR measurements on total RNA for ie1 (**A**), the early genes m152 (**B**) and m169 (**C**) as well as for the late genes m129/131 (**D**) and M94 (**E**). Total RNA rates were normalized to Lbr expression. Shown are the combined data (means +/− SD) of three independent experiments.
(TIF)

Figure S5 **Regulation of viral gene expression following low and high MOI.** NIH-3T3 cells were infected with (**A**) an MOI of 0.5 (low MOI) and (**B**) an MOI of 10 (high MOI, data taken from Figure 5B–F). Newly transcribed RNA was labeled from −1 to 0 (mock), 1–2, 3–4 and 5–6 hpi. Following purification of newly transcribed RNA, expression levels of ie1, m152, m169, m129/131 and M94 were determined by qRT-PCR normalized for Lbr.
(TIF)

Figure S6 **Classification of reads aligned to mouse transcripts and genomic sequence.** Reads were classified according to their alignment position in mouse transcripts or genomic sequence as exon, intron, exon-exon junction, or exon-intron junction reads. Shown are the relative frequencies of each class for total, pre-existing, and newly transcribed RNA samples derived from NIH-3T3 cells at various times of infection. The high fraction of intronic reads in newly transcribed RNA samples is consistent with the increased proportion of premature transcripts present in newly transcribed RNA.
(TIF)

Figure S7 **RNA-seq data on MCMV gene expression using total RNA and unlabeled pre-existing RNA.** Shown are the read coverages of all viral genes across the whole MCMV genome obtained by RNA-seq for uninfected cells for (**A**) newly transcribed RNA samples and 25 hpi and 48 hpi for (**B**) total RNA samples and (**C**) pre-existing RNA samples normalized to the total number of mapped exonic mouse reads. Positions of exemplary genes as well as representative gene clusters showing concordant regulation are indicated; SCP = small capsid protein (m48.2), POL = polymerase (M54). Blue represent reads matching to the direct DNA strand and red represents reads matching to the complementary DNA strand.
(TIF)

Table S1 **A. List of MCMV-regulated cellular genes.** This table contains all (n = 1,674) cellular genes significantly (p<0.05) regulated by >2-fold in at least one condition (total or newly transcribed RNA) during the first six hours of MCMV infection compared to uninfected NIH-3T3 cells. Log_2_ values of detected fold-changes are depicted. Positive values represent up-regulation, negative represent down-regulation. **B. List of MCMV-regulated genes with greater change in total RNA than in newly transcribed RNA.** This table contains all genes which showed at least 1.41-fold (2^0.5^) greater regulation in total RNA than in newly transcribed RNA at any time point of infection (0–6 hpi). Log_2_ values of the fold-changes compared to uninfected cells are shown. Only three genes (Mela, Tk1 and Plk1, indicated in yellow) showed >2-fold stronger regulation in total RNA than in newly transcribe RNA, arguing against a significant contribution of changes in RNA decay rates contributing to the observed changes. **C. Clusters of genes regulated with distinct kinetics.** Genes significantly regulated during the first 6 hours of lytic MCMV infection were grouped into clusters showing distinct kinetics of regulation. Genes belonging to the five clusters we identified are shown. Log_2_ values of changes compared to mock are depicted. Positive values represent up-regulation, negative represent down-regulation. Below each cluster, the filters applied are shown highlighted in red.
(XLSX)

Table S2 **Bioinformatic analysis on MCMV-regulated gene clusters.** **A. Enrichment analysis of Gene Ontology ‘Biological Process’ terms and KEGG pathways.** Results of the Gene Ontology (GO) (‘Biological Process’ only) and KEGG pathway enrichment analysis. For significantly enriched annotation categories (p-value<0.01) the count (i.e. number of genes involved in the term), percentage (involved genes/total genes), and a modified Fisher Exact p-value are given. Moreover, genes annotated to each category are listed with their Affymetrix identifiers. **B. TFM Explorer results** Over-represented transcription factor binding sites (TFBSs) for each cluster were predicted using Transcription Factor Matrix (TFM) Explorer. See methods for scanning parameters. Identified TFBSs are given with their rank (most significant first); matrix name; transcription factor name; information of content of the matrix; gc content of the matrix (G+C)%; location of the identified binding sites (relatively to TSS); and p-value. For each identified TFBS the entire list of hits in all input sequences is also given. Information includes gene name; location (position and strand orientation) of binding motif; matrix similarity; and motif instance.
(XLSX)

Table S3 **List of PCR Primers.** All PCR primers used in this study are listed.
(XLSX)

Table S4 **Analysis of RNA-seq data.** **A. Statistics of RNA-seq data.** Number of reads from MCMV infected NIH-3T3 cells aligned to the cellular and viral genome. Shown are (I) the number of reads per sample, (II,III) intron, exon and junction reads of cellular (II) and viral genes (III). **B. RPKM values of viral transcripts.** For all MCMV genes reads mapped to the annotated coding sequences of Smith strain MCMV were used for RPKM calculation. Normalization for sample size was done using the total number of reads mapped to exons or exon-exon junctions in mouse or virus. RPKM values are rounded.
(XLSX)
