# Supplementary figures and images for: Real-time Transcriptional Profiling of Cellular and Viral Gene Expression during Lytic Cytomegalovirus Infection
Source: PLoS Pathog. 2012 Sep 6;8(9):e1002908. doi: 10.1371/journal.ppat.1002908 (PMC3435240; doi:10.1371/journal.ppat.1002908)

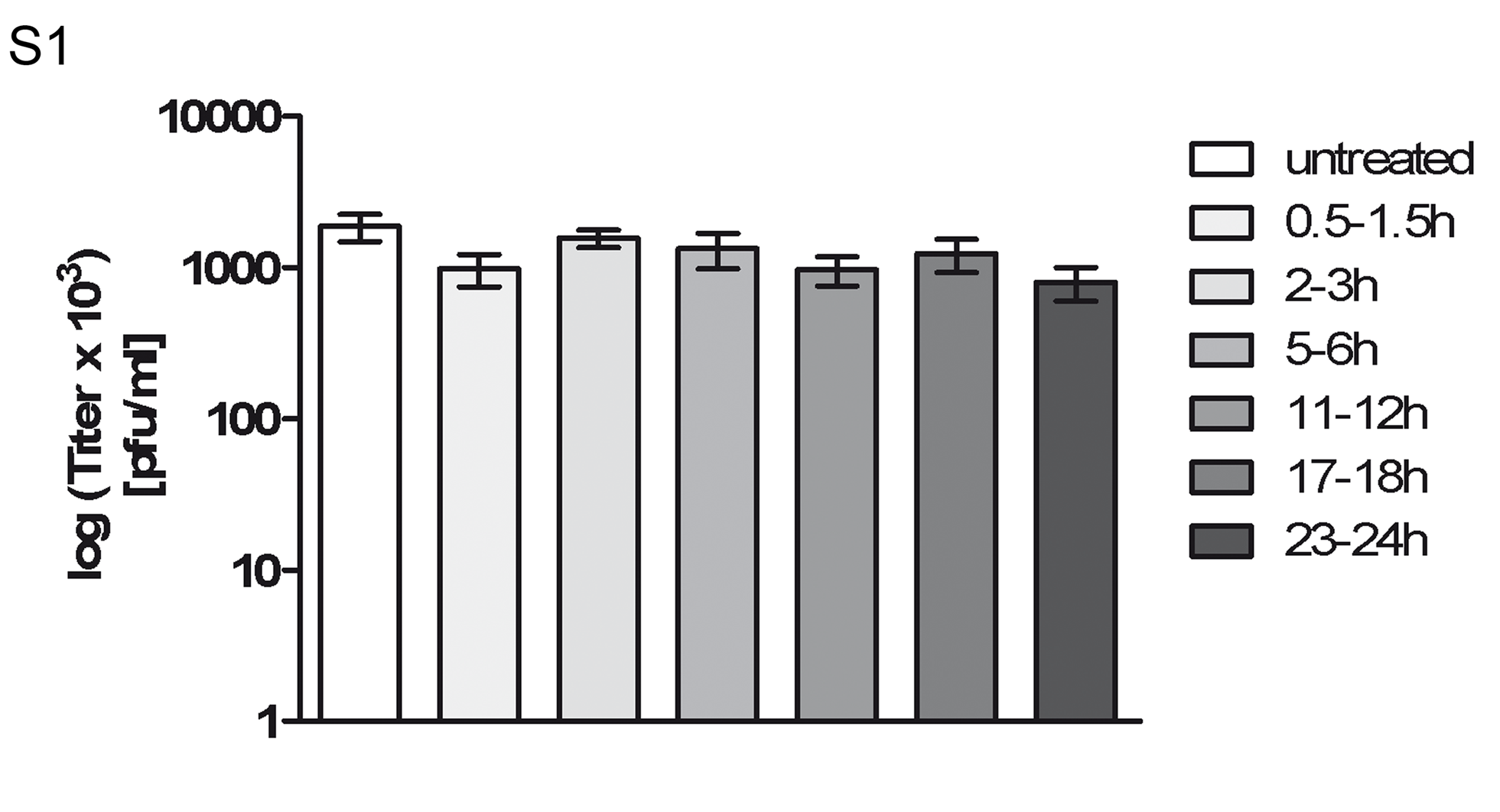

Supplement: Figure S1 — No effect of 1 h 200 µM 4sU treatment on virus replication. NIH-3T3 fibroblasts were infected with MCMV at an MOI of 10 for 48 h. Samples were exposed to 200 µM 4sU for 1 h at different time points of infection or left untreated. Supernatants were harvested and titrated at 48 hpi. Shown are the means +/− SD of three biological replicates. (TIF) [file ppat.1002908.s001.tif]

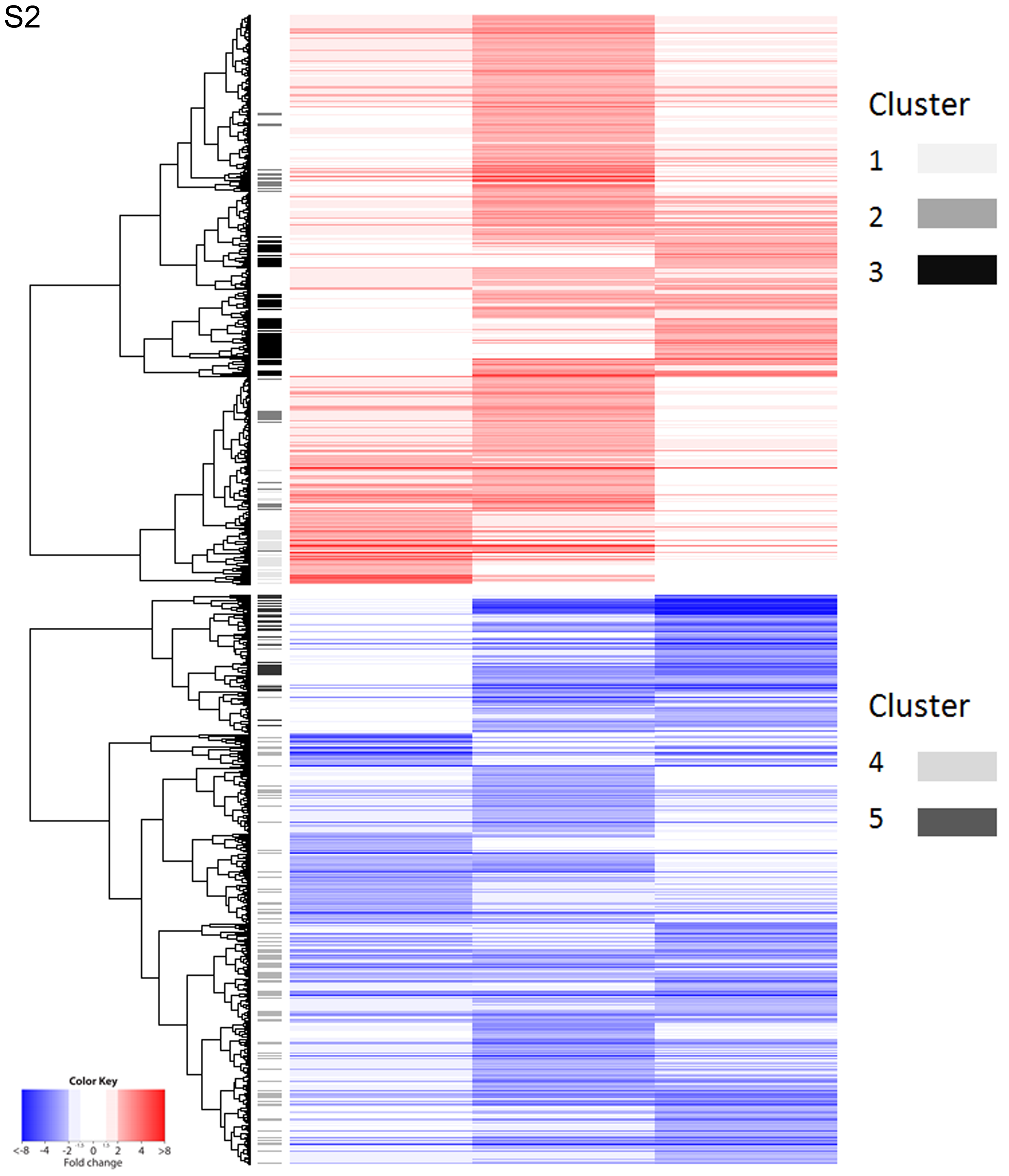

Supplement: Figure S2 — Cluster of all differentially regulated genes in newly transcribed RNA. Heat-map indicating fold-changes with rows representing genes and columns representing time points post infection. Red represents up-regulation, blue down-regulation (>2-fold, p≤0.05) in newly transcribed RNA relative to uninfected cells. Ordering of genes was determined using non-supervised hierarchical clustering across two conditions: regulation at 3–4 hpi minus 1–2 hpi and regulation at 5–6 hpi minus 1–2 hpi (using log2-values of fold-changes compared to uninfected cells). Clustering was performed separately for induced and repressed genes. Gray scales depicted on the left mark genes belonging to the corresponding clusters described in Figure 2A. (TIF) [file ppat.1002908.s002.tif]

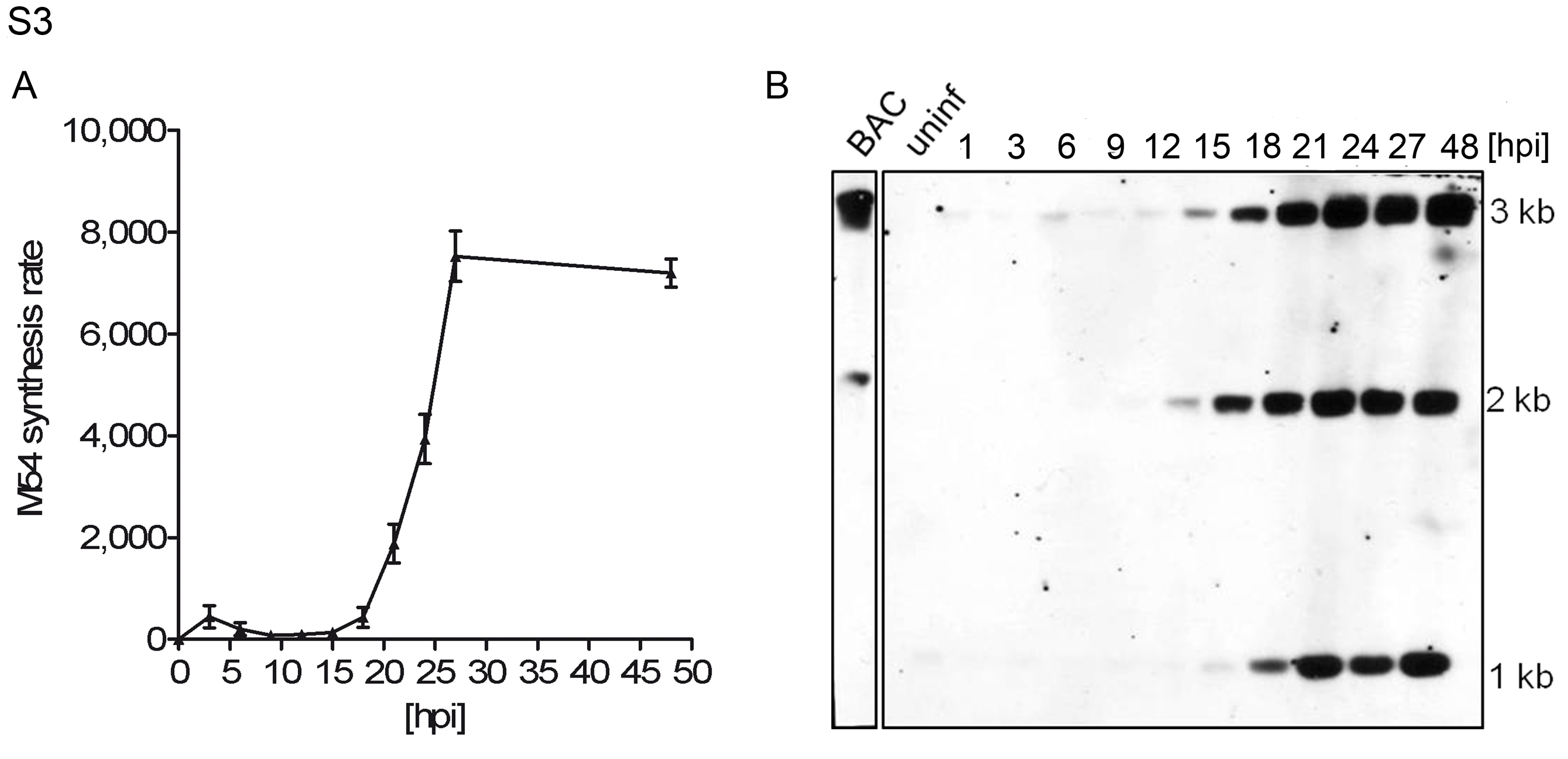

Supplement: Figure S3 — Temporal kinetics of MCMV replication. NIH-3T3 cells were infected with MCMV at an MOI of 10 and DNA was isolated at various times of infection using the DNeasy Blood & Tissue Kit (Qiagen) according to the manufacturer's instructions. Prior to amplification, extracted DNA was digested with PaeI for 1 h, 37°C followed by heat inactivation. (A) TaqMan qRT-PCR was performed in triplicates for MCMV M54 and cellular Lbr using the ABI Prism 7700 sequence detector (Applied Biosystems) as described [103]. Synthesis rates were normalized to Lbr. Shown are the means +/− SD of three independent experiments. (B) Southern Blot analysis was performed as described in Popa et al. [104]. Shown is the detection of different genome fragments in DNA isolated from NIH-3T3 cells at various times of infection. MCMV BAC-DNA (BAC) served as a negative control. The 3 kb fragment serves as a measurement of genomic load, the 2 kb fragment for concatameric/circular DNA and the 1 kb fragment for cleaved genomic DNA. Due to its circular form, digested BAC DNA produces only two of the three fragments. (TIF) [file ppat.1002908.s003.tif]

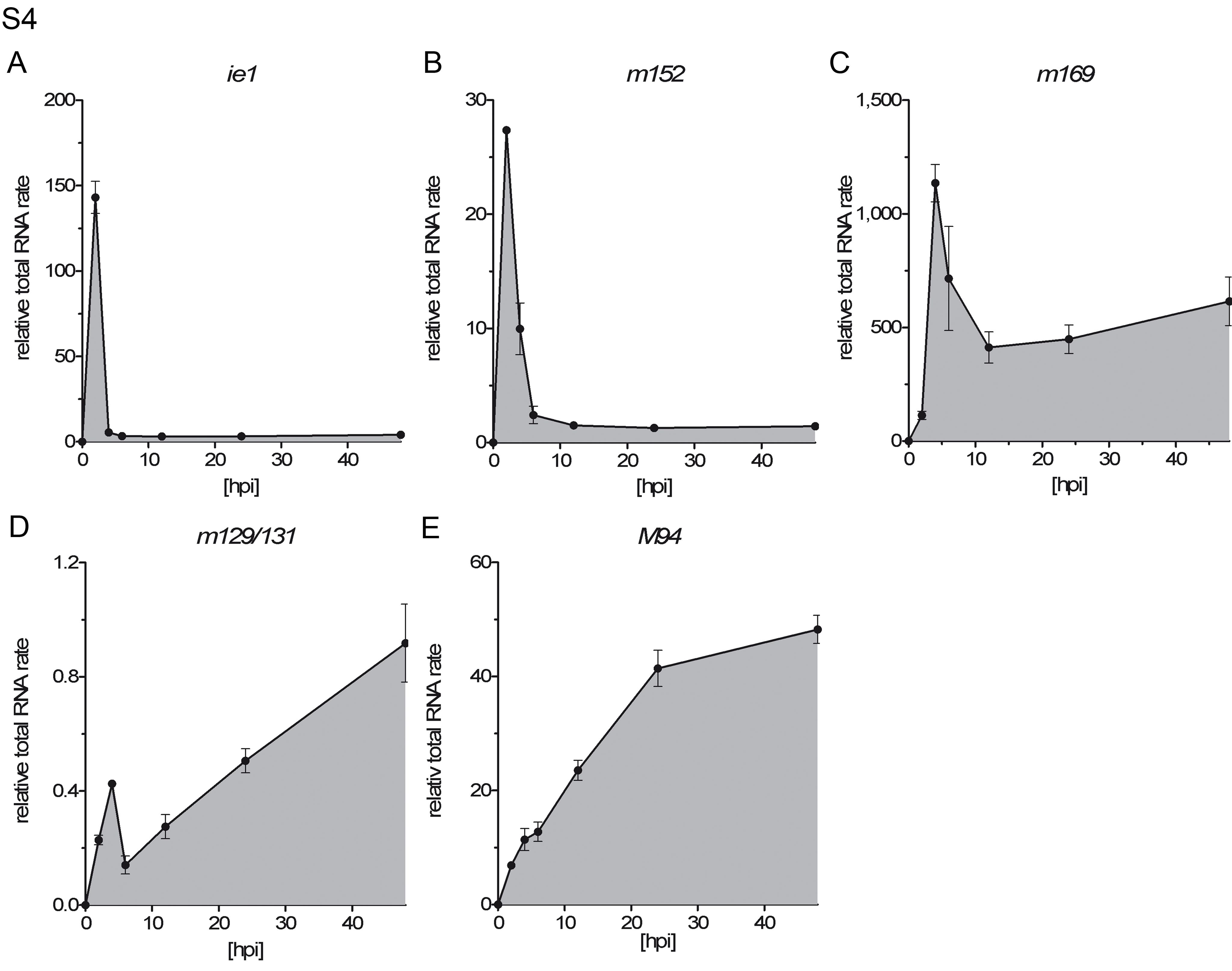

Supplement: Figure S4 — Gene expression kinetics of exemplary viral genes in total RNA. Shown are qRT-PCR measurements on total RNA for ie1 (A), the early genes m152 (B) and m169 (C) as well as for the late genes m129/131 (D) and M94 (E). Total RNA rates were normalized to Lbr expression. Shown are the combined data (means +/− SD) of three independent experiments. (TIF) [file ppat.1002908.s004.tif]

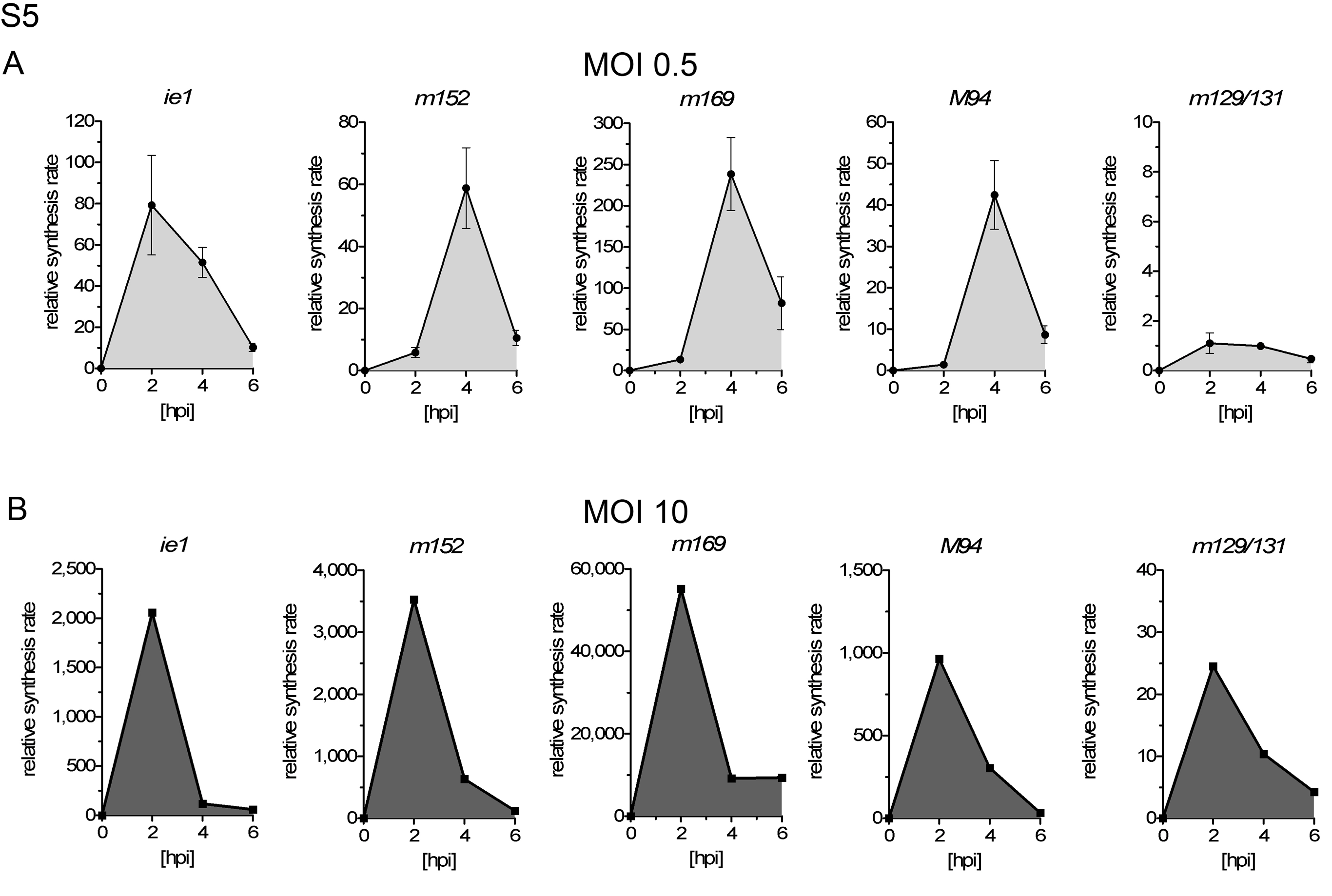

Supplement: Figure S5 — Regulation of viral gene expression following low and high MOI. NIH-3T3 cells were infected with (A) an MOI of 0.5 (low MOI) and (B) an MOI of 10 (high MOI, data taken from Figure 5B–F). Newly transcribed RNA was labeled from −1 to 0 (mock), 1–2, 3–4 and 5–6 hpi. Following purification of newly transcribed RNA, expression levels of ie1, m152, m169, m129/131 and M94 were determined by qRT-PCR normalized for Lbr. (TIF) [file ppat.1002908.s005.tif]

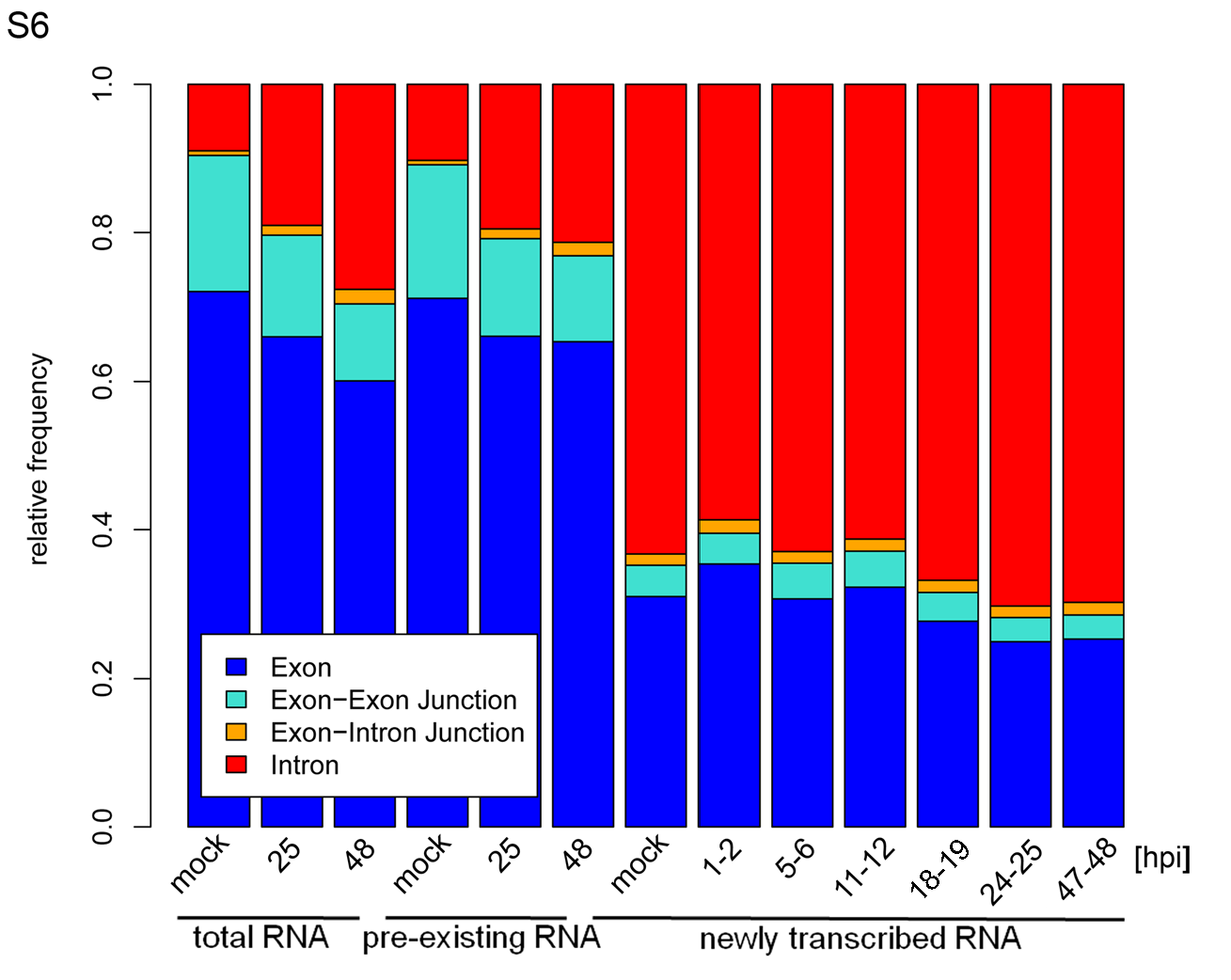

Supplement: Figure S6 — Classification of reads aligned to mouse transcripts and genomic sequence. Reads were classified according to their alignment position in mouse transcripts or genomic sequence as exon, intron, exon-exon junction, or exon-intron junction reads. Shown are the relative frequencies of each class for total, pre-existing, and newly transcribed RNA samples derived from NIH-3T3 cells at various times of infection. The high fraction of intronic reads in newly transcribed RNA samples is consistent with the increased proportion of premature transcripts present in newly transcribed RNA. (TIF) [file ppat.1002908.s006.tif]

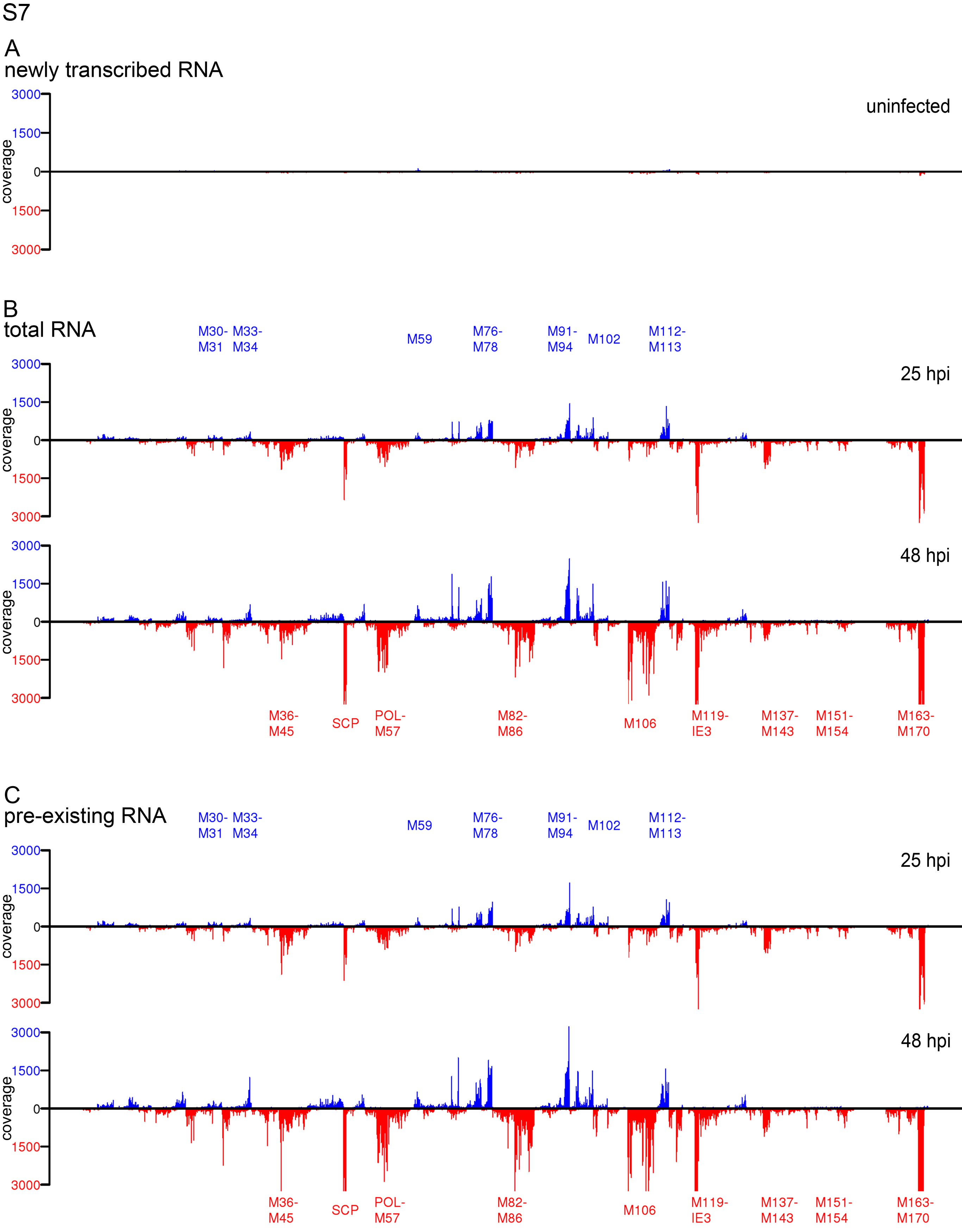

Supplement: Figure S7 — RNA-seq data on MCMV gene expression using total RNA and unlabeled pre-existing RNA. Shown are the read coverages of all viral genes across the whole MCMV genome obtained by RNA-seq for uninfected cells for (A) newly transcribed RNA samples and 25 hpi and 48 hpi for (B) total RNA samples and (C) pre-existing RNA samples normalized to the total number of mapped exonic mouse reads. Positions of exemplary genes as well as representative gene clusters showing concordant regulation are indicated; SCP = small capsid protein (m48.2), POL = polymerase (M54). Blue represent reads matching to the direct DNA strand and red represents reads matching to the complementary DNA strand. (TIF) [file ppat.1002908.s007.tif]
